# 4-{(*E*)-[2-(4-Iodo­but­oxy)benzyl­idene]amino}-1,5-dimethyl-2-phenyl-1*H*-pyrazol-3(2*H*)-one

**DOI:** 10.1107/S1600536810020374

**Published:** 2010-06-05

**Authors:** Hoong-Kun Fun, Madhukar Hemamalini, Abdullah M. Asiri, Salman A. Khan

**Affiliations:** a X-ray Crystallography Unit, School of Physics, Universiti Sains Malaysia, 11800 USM, Penang, Malaysia; b Department of Chemistry, Faculty of Science, King Abdu Aziz University, Jeddah, Saudi Arabia

## Abstract

The title Schiff base compound, C_22_H_24_IN_3_O_2_, adopts an *E* configuration about the central C=N bond. The pyrazolone ring makes a dihedral angle of 49.68 (10)° with its attached phenyl ring. The phenolate plane makes dihedral angles of 16.78 (9) and 50.54 (9)°, respectively, with the pyrazolone ring and the terminal phenyl ring. An intra­molecular C—H⋯O hydrogen bond generates an *S*(6) ring motif. In the crystal structure, an inter­molecular C—H⋯O hydrogen bond is also observed.

## Related literature

For background to and applications of Schiff bases, see: Tarafder *et al.* (2002 ▶); Silver & Soderlund (2005 ▶); Vicini *et al.* (2003 ▶); Ozdemir *et al.* (2007 ▶); Joshi *et al.* (2004 ▶). For background to and the biological activity of 4-amino­anti­pyrene and its derivatives, see: Jain *et al.* (2003 ▶); Filho *et al.* (1998 ▶); Sondhi *et al.* (1999 ▶); Mishra (1999 ▶); Sondhi *et al.* (2001 ▶). For related structures, see: Eryigit & Kendi (1998 ▶); Manikandan *et al.* (2000 ▶). For hydrogen-bond motifs, see: Bernstein *et al.* (1995 ▶). For the stability of the temperature controller used in the data collection, see: Cosier & Glazer (1986 ▶).
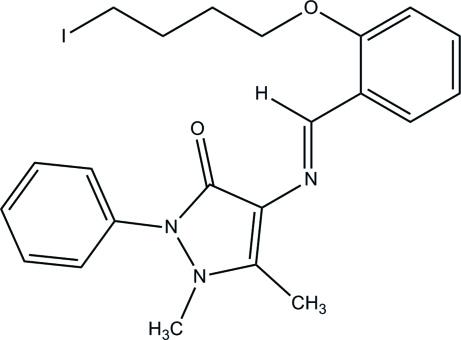


## Experimental

### 

#### Crystal data

C_22_H_24_IN_3_O_2_
*M* *_r_* = 489.34
Monoclinic, 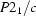

*a* = 11.5235 (10) Å
*b* = 16.4156 (14) Å
*c* = 11.2828 (9) Å
β = 94.010 (2)°
*V* = 2129.1 (3) Å^3^
*Z* = 4
Mo *K*α radiation
μ = 1.53 mm^−1^
*T* = 100 K
0.41 × 0.34 × 0.29 mm

#### Data collection

Bruker APEXII DUO CCD area-detector diffractometer
Absorption correction: multi-scan (*SADABS*; Bruker, 2009 ▶) *T*_min_ = 0.571, *T*_max_ = 0.663
36214 measured reflections
9632 independent reflections
7935 reflections with *I* > 2σ(*I*)
*R*_int_ = 0.025

#### Refinement

*R*[*F* ^2^ > 2σ(*F* ^2^)] = 0.042
*wR*(*F* ^2^) = 0.159
*S* = 1.05
9632 reflections
255 parameters
H-atom parameters constrained
Δρ_max_ = 1.26 e Å^−3^
Δρ_min_ = −1.68 e Å^−3^

### 

Data collection: *APEX2* (Bruker, 2009 ▶); cell refinement: *SAINT* (Bruker, 2009 ▶); data reduction: *SAINT*; program(s) used to solve structure: *SHELXTL* (Sheldrick, 2008 ▶); program(s) used to refine structure: *SHELXTL*; molecular graphics: *SHELXTL*; software used to prepare material for publication: *SHELXTL* and *PLATON* (Spek, 2009 ▶).

## Supplementary Material

Crystal structure: contains datablocks global, I. DOI: 10.1107/S1600536810020374/is2554sup1.cif

Structure factors: contains datablocks I. DOI: 10.1107/S1600536810020374/is2554Isup2.hkl

Additional supplementary materials:  crystallographic information; 3D view; checkCIF report

## Figures and Tables

**Table 1:** Hydrogen-bond geometry (Å, °)

*D*—H⋯*A*	*D*—H	H⋯*A*	*D*⋯*A*	*D*—H⋯*A*
C10—H10*A*⋯O1	0.93	2.30	2.995 (2)	132
C17—H17*B*⋯O1^i^	0.97	2.42	3.193 (2)	137

Symmetry code: (i) 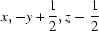.

**Fig. 1.**
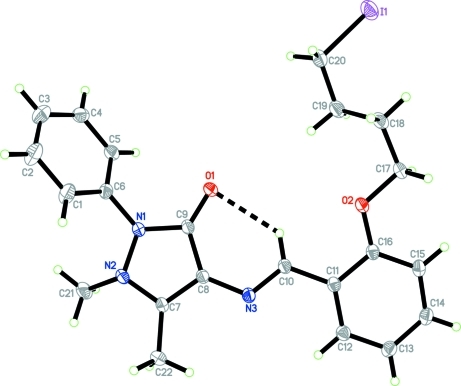
The asymmetric unit of the title compound, showing 50% probability displacement ellipsoids and the atom-numbering scheme. An intramolecular hydrogen bond is shown as dashed line.

**Fig. 2.**
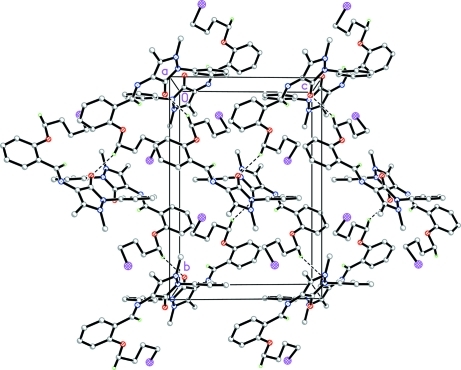
The crystal packing of the title compound, showing hydrogen-bonded (dashed lines) network. H atoms not involved in the hydrogen bond interactions are omitted for clarity.

## supplementary crystallographic information

### Comment

Schiff bases are generally synthesized from the condensation of primary amines and active carbonyl group. Various heterocyclic ring containing Schiff bases were reported to possess cytotoxic (Tarafder *et al.*, 2002), anticonvulsant (Silver & Soderlund, 2005), antiproliferative (Vicini *et al.*, 2003), anticancer and antifungal activities (Ozdemir *et al.*, 2007). It's also used as ligands for the complexes synthesis (Joshi *et al.*, 2004). As evident from the literature, it was noted that a lot of research has been carried out on Schiff bases but no work has been done on the long chain Schiff base. 4-Aminoantipyrene, which contain pyrazolone ring, is an important compound in the class analgesic agent in otic solutions in combination with other analgesic such as benzocaine and phenylephrine. Pyrazolone is a five-membered lactam ring compound containing two N atoms and ketone in the same molecule. Lactam structure is an active nucleus in pharmacological activity, especially in the class of nonsteroidal antiinflammatory agents used in the treatment of arthritis and other musculo skeletal and joint disorders. Pyrazolone derivatives, as lactam structure related compounds, are also widely used in preparing dyes and pigments. 4-Aminoantipyrene and its derivatives have potential biological activities (Jain *et al.*, 2003). Analgesic and antiinflammatory activities of the 4-aminoantipyrene complexes were extensively studied and reported (Filho *et al.*, 1998; Sondhi *et al.*, 1999). Apart from that, antimicrobial and anticancer activity of the 4-aminoantipyrine derivatives and their metal complexes caught the attention of many researchers during last decade (Mishra, 1999; Sondhi *et al.*, 2001). In this paper we report the synthesis and the crystal structure of a mono Schiff base bearing butyl iodide side chain. It is noteworthy that the alkylating agent used in this reaction is dibromo butyl, and after obtaining the *O*-alkylation product, the charge transfer catalyst used caused the free bromide atom to be substituted by an iodide atom.

The title compound (I) is shown in Fig. 1. The molecule adopts a trans configuration about the central C10═N3 double bond.  The C—N bond lengths of N1—C6 [1.422 (2) Å], N1—C9 [1.398 (2) Å], N2—C21 [1.459 (3) Å], N2—C7 [1.365 (2) Å] and N3—C8 [1.389 (2) Å] are normal for C—N single-bond distances. The distance between C10—N3 [1.290 (2) Å] is typical for a C═ N double-bond distance. These bonds are comparable with those in *N*-(1*H*-benzoimidazol-2-ylmethyl)-*N*-(2,6-dichlorophenyl) amine (Eryigit & Kendi, 1998). The N1—N2 [1.403 (2) Å] single-bond length is comparable with that in 2,6-bis(3,5-dimethylpyrazol-1-ylmethyl)pyridine (Manikandan *et al.*, 2000).

Atom O1 deviates from the pyrazoline mean plane by 0.028 (1) Å. The pyrazolone ring (C7–C9/N1/N2) is almost planar, with maximum deviation of 0.045 (2) Å for atom N2. It makes a dihedral angle of 49.68 (10)° with its attached phenyl ring (C1–C6). The phenolate residue (C11–C16/O2) is essentially planar, with maximum deviation of 0.031 (2) Å for O2. This plane makes dihedral angles of 16.78 (9) and 50.54 (9)°, respectively, with the pyrazolone ring (C7–C9/N1/N2) and the terminal (C1–C6) phenyl ring. The N2-N1-C6-C5 and C1-C6-N1-C9 torsion angles are -147.45 (18) and -116.1 (2)°, respectively.

In the crystal structure (Fig. 2), intramolecular C10—H10A···O1 hydrogen bond interactions generate an *S*(6) ring motif (Bernstein *et al.*, 1995). The crystal packing is consolidated by weak non-classical intermolecular C17—H17B···O1 hydrogen bonds (Table 1). The combination of both intra and intermolecular C—H···O hydrogen bonds stabilize the crystal structure. There exists an unusual short contact between atoms I1 and C8 with a distance of 3.3606 (17) Å, which is shorter than the sum of their van der Waals radii.

### Experimental

The title compound was synthesized by the reaction of mono Schiff base (1 g, 0.0032 mol) with dibromo butane (0.0016 mol) in the presence of freshly heated K_2_CO_3_ (0.0097 mol) and tetrabutylamonium iodide (PTC) (0.0004 mol) in dry acetone with continuous stirring at 40 °C for 8h. After the completion of the reaction, the product obtained was purified by passing through silica-gel column (60-120 mesh) and further crystallized from methanol. Yield: 65 %; m. p. 136 °C. IR (KBr) *ν*_max_ cm^-1^: 3014 (C–H aromatic), 1666 (C═O), 1571 (HC═N), 1299 (C–O), 1108 (C–N). ^1^H-NMR (CDCl_3_) *δ*: 10.13 (s, 1H, C–H olefinic), 8.22-6.96 (m, 9H, C–H aromatic), 3.57 (s, O–CH_2_CH_2_), 3.33 (s, N–CH_3_), 2.92 (s, I–CH_2_), 2.22 (s,-CH_3_), 2.11 (s, 2×CH_2_).

### Refinement

All hydrogen atoms were positioned geometrically (C–H = 0.93–0.97 Å) and were refined using a riding model, with *U*_iso_(H) = 1.2 or 1.5*U*_eq_(C). A rotating group model was used for the methyl group. The highest peak of 1.26 eÅ^3^ was found at a distance of 0.70 Å from I1 and the deepest hole of -1.68 eÅ^3^ was at a distance of 0.54 Å from I1.

### Crystal data

**Table:**

C_22_H_24_IN_3_O_2_	*F*(000) = 984
*M**_r_* = 489.34	*D*_x_ = 1.527 Mg m^−^^3^
Monoclinic, *P*2_1_/*c*	Mo *K*α radiation, λ = 0.71073 Å
Hall symbol: -P 2ybc	Cell parameters from 9944 reflections
*a* = 11.5235 (10) Å	θ = 2.7–35.4°
*b* = 16.4156 (14) Å	µ = 1.53 mm^−^^1^
*c* = 11.2828 (9) Å	*T* = 100 K
β = 94.010 (2)°	Blcok, yellow
*V* = 2129.1 (3) Å^3^	0.41 × 0.34 × 0.29 mm
*Z* = 4	

### Data collection

**Table:**

Bruker APEXII DUO CCD area-detector diffractometer	9632 independent reflections
Radiation source: fine-focus sealed tube	7935 reflections with *I* > 2σ(*I*)
graphite	*R*_int_ = 0.025
φ and ω scans	θ_max_ = 35.6°, θ_min_ = 2.2°
Absorption correction: multi-scan (*SADABS*; Bruker, 2009)	*h* = −16→18
*T*_min_ = 0.571, *T*_max_ = 0.663	*k* = −26→26
36214 measured reflections	*l* = −17→18

### Refinement

**Table:**

Refinement on *F*^2^	Primary atom site location: structure-invariant direct methods
Least-squares matrix: full	Secondary atom site location: difference Fourier map
*R*[*F*^2^ > 2σ(*F*^2^)] = 0.042	Hydrogen site location: inferred from neighbouring sites
*wR*(*F*^2^) = 0.159	H-atom parameters constrained
*S* = 1.05	*w* = 1/[σ^2^(*F*_o_^2^) + (0.1027*P*)^2^ + 1.5532*P*] where *P* = (*F*_o_^2^ + 2*F*_c_^2^)/3
9632 reflections	(Δ/σ)_max_ = 0.001
255 parameters	Δρ_max_ = 1.26 e Å^−^^3^
0 restraints	Δρ_min_ = −1.68 e Å^−^^3^

### Special details

**Table:**

Experimental. The crystal was placed in the cold stream of an Oxford Cryosystems Cobra open-flow nitrogen cryostat (Cosier & Glazer, 1986) operating at 100.0 (1) K.
Geometry. All s.u.'s (except the s.u. in the dihedral angle between two l.s. planes) are estimated using the full covariance matrix. The cell s.u.'s are taken into account individually in the estimation of s.u.'s in distances, angles and torsion angles; correlations between s.u.'s in cell parameters are only used when they are defined by crystal symmetry. An approximate (isotropic) treatment of cell s.u.'s is used for estimating s.u.'s involving l.s. planes.
Refinement. Refinement of F^2^ against ALL reflections. The weighted R-factor wR and goodness of fit S are based on F^2^, conventional R-factors R are based on F, with F set to zero for negative F^2^. The threshold expression of F^2^ > 2σ(F^2^) is used only for calculating R-factors(gt) etc. and is not relevant to the choice of reflections for refinement. R-factors based on F^2^ are statistically about twice as large as those based on F, and R- factors based on ALL data will be even larger.

### Fractional atomic coordinates and isotropic or equivalent isotropic displacement parameters (Å^2^)

**Table:**

	*x*	*y*	*z*	*U*_iso_*/*U*_eq_	
I1	0.290561 (14)	0.362436 (10)	0.802978 (17)	0.03475 (7)	
O1	0.68399 (13)	0.05872 (8)	0.94462 (12)	0.0192 (2)	
O2	0.71122 (14)	0.22745 (9)	0.66181 (13)	0.0206 (2)	
N1	0.76696 (14)	−0.05557 (10)	1.03829 (14)	0.0182 (3)	
N2	0.87138 (14)	−0.09878 (10)	1.03161 (15)	0.0200 (3)	
N3	0.89080 (14)	0.03071 (9)	0.78003 (13)	0.0163 (2)	
C1	0.78638 (19)	−0.05051 (15)	1.25448 (18)	0.0266 (4)	
H1A	0.8669	−0.0531	1.2531	0.032*	
C2	0.7339 (2)	−0.04626 (17)	1.36239 (19)	0.0315 (5)	
H2A	0.7798	−0.0461	1.4336	0.038*	
C3	0.6135 (2)	−0.04230 (14)	1.3637 (2)	0.0275 (4)	
H3A	0.5791	−0.0395	1.4357	0.033*	
C4	0.54450 (18)	−0.04253 (12)	1.25739 (19)	0.0232 (3)	
H4A	0.4640	−0.0392	1.2586	0.028*	
C5	0.59526 (17)	−0.04769 (11)	1.14967 (18)	0.0199 (3)	
H5A	0.5492	−0.0490	1.0786	0.024*	
C6	0.71592 (17)	−0.05082 (11)	1.14923 (16)	0.0186 (3)	
C7	0.92082 (16)	−0.07340 (11)	0.93152 (15)	0.0172 (3)	
C8	0.85924 (15)	−0.00877 (10)	0.88180 (15)	0.0156 (3)	
C9	0.75980 (16)	0.00540 (10)	0.95158 (15)	0.0161 (3)	
C10	0.82737 (16)	0.09014 (10)	0.73798 (15)	0.0166 (3)	
H10A	0.7665	0.1092	0.7803	0.020*	
C11	0.85006 (16)	0.12799 (10)	0.62451 (15)	0.0160 (3)	
C12	0.93106 (16)	0.09552 (11)	0.55089 (16)	0.0184 (3)	
H12A	0.9768	0.0516	0.5775	0.022*	
C13	0.94457 (18)	0.12744 (12)	0.43891 (17)	0.0209 (3)	
H13A	0.9995	0.1057	0.3912	0.025*	
C14	0.87488 (18)	0.19228 (12)	0.39885 (16)	0.0210 (3)	
H14A	0.8814	0.2124	0.3226	0.025*	
C15	0.79573 (17)	0.22751 (11)	0.47051 (16)	0.0195 (3)	
H15A	0.7510	0.2717	0.4432	0.023*	
C16	0.78371 (16)	0.19599 (10)	0.58414 (15)	0.0167 (3)	
C17	0.64112 (18)	0.29607 (12)	0.62471 (18)	0.0224 (3)	
H17A	0.5886	0.2813	0.5572	0.027*	
H17B	0.6902	0.3403	0.6010	0.027*	
C18	0.57265 (18)	0.32256 (11)	0.72692 (19)	0.0229 (3)	
H18A	0.5220	0.3673	0.7010	0.027*	
H18B	0.6262	0.3426	0.7905	0.027*	
C19	0.49946 (19)	0.25498 (12)	0.7752 (2)	0.0244 (3)	
H19A	0.4505	0.2319	0.7103	0.029*	
H19B	0.5506	0.2122	0.8072	0.029*	
C20	0.4233 (2)	0.28321 (15)	0.8710 (2)	0.0301 (4)	
H20A	0.4709	0.3111	0.9327	0.036*	
H20B	0.3884	0.2362	0.9066	0.036*	
C21	0.8675 (2)	−0.18482 (14)	1.0635 (2)	0.0313 (5)	
H21A	0.9438	−0.2080	1.0613	0.047*	
H21B	0.8147	−0.2129	1.0080	0.047*	
H21C	0.8414	−0.1902	1.1421	0.047*	
C22	1.02298 (18)	−0.11555 (13)	0.88815 (18)	0.0223 (3)	
H22A	1.0828	−0.1196	0.9515	0.033*	
H22B	1.0517	−0.0852	0.8236	0.033*	
H22C	1.0008	−0.1692	0.8612	0.033*	

### Atomic displacement parameters (Å^2^)

**Table:**

	*U*^11^	*U*^22^	*U*^33^	*U*^12^	*U*^13^	*U*^23^
I1	0.02522 (9)	0.03191 (10)	0.04766 (12)	0.00818 (5)	0.00638 (7)	0.01372 (6)
O1	0.0219 (6)	0.0172 (5)	0.0185 (5)	0.0063 (4)	0.0021 (4)	0.0012 (4)
O2	0.0269 (7)	0.0163 (5)	0.0188 (5)	0.0067 (5)	0.0033 (5)	0.0036 (4)
N1	0.0186 (6)	0.0191 (6)	0.0169 (6)	0.0048 (5)	0.0018 (5)	0.0040 (5)
N2	0.0181 (6)	0.0195 (6)	0.0224 (7)	0.0051 (5)	0.0023 (5)	0.0069 (5)
N3	0.0184 (6)	0.0155 (6)	0.0147 (6)	0.0014 (5)	−0.0003 (4)	0.0013 (4)
C1	0.0213 (8)	0.0392 (11)	0.0191 (8)	−0.0067 (7)	0.0002 (6)	0.0047 (7)
C2	0.0296 (10)	0.0457 (13)	0.0192 (8)	−0.0092 (9)	0.0020 (7)	0.0032 (8)
C3	0.0321 (10)	0.0283 (9)	0.0232 (8)	−0.0054 (8)	0.0091 (7)	−0.0004 (7)
C4	0.0233 (8)	0.0184 (7)	0.0288 (9)	0.0010 (6)	0.0076 (7)	0.0021 (6)
C5	0.0197 (7)	0.0165 (7)	0.0234 (8)	0.0014 (5)	0.0012 (6)	0.0012 (6)
C6	0.0200 (7)	0.0182 (7)	0.0177 (7)	−0.0004 (5)	0.0020 (5)	0.0031 (5)
C7	0.0181 (7)	0.0169 (6)	0.0162 (6)	0.0029 (5)	−0.0003 (5)	0.0028 (5)
C8	0.0174 (6)	0.0146 (6)	0.0145 (6)	0.0020 (5)	−0.0001 (5)	0.0010 (5)
C9	0.0190 (7)	0.0143 (6)	0.0149 (6)	0.0017 (5)	−0.0002 (5)	0.0008 (5)
C10	0.0206 (7)	0.0139 (6)	0.0151 (6)	0.0016 (5)	0.0005 (5)	0.0006 (5)
C11	0.0187 (7)	0.0137 (6)	0.0153 (6)	−0.0002 (5)	−0.0008 (5)	0.0002 (5)
C12	0.0204 (7)	0.0184 (7)	0.0162 (6)	0.0021 (6)	−0.0001 (5)	−0.0001 (5)
C13	0.0223 (8)	0.0235 (8)	0.0169 (7)	−0.0005 (6)	0.0027 (6)	0.0009 (6)
C14	0.0227 (8)	0.0232 (8)	0.0169 (7)	−0.0017 (6)	0.0007 (6)	0.0042 (6)
C15	0.0209 (7)	0.0187 (7)	0.0186 (7)	−0.0012 (6)	−0.0009 (6)	0.0050 (5)
C16	0.0199 (7)	0.0138 (6)	0.0162 (6)	−0.0010 (5)	−0.0001 (5)	0.0006 (5)
C17	0.0257 (8)	0.0170 (7)	0.0245 (8)	0.0053 (6)	0.0026 (6)	0.0055 (6)
C18	0.0246 (8)	0.0137 (6)	0.0304 (9)	0.0020 (6)	0.0024 (7)	0.0009 (6)
C19	0.0252 (8)	0.0172 (7)	0.0310 (9)	0.0018 (6)	0.0025 (7)	0.0036 (6)
C20	0.0298 (10)	0.0298 (10)	0.0314 (10)	0.0106 (8)	0.0063 (8)	0.0122 (8)
C21	0.0305 (10)	0.0227 (9)	0.0418 (12)	0.0084 (8)	0.0110 (9)	0.0154 (8)
C22	0.0210 (8)	0.0219 (7)	0.0243 (8)	0.0062 (6)	0.0031 (6)	0.0024 (6)

### Geometric parameters (Å, °)

**Table:**

I1—C20	2.111 (2)	C11—C12	1.398 (3)
O1—C9	1.235 (2)	C11—C16	1.410 (2)
O2—C16	1.355 (2)	C12—C13	1.387 (3)
O2—C17	1.432 (2)	C12—H12A	0.9300
N1—C9	1.398 (2)	C13—C14	1.390 (3)
N1—N2	1.403 (2)	C13—H13A	0.9300
N1—C6	1.422 (2)	C14—C15	1.387 (3)
N2—C7	1.365 (2)	C14—H14A	0.9300
N2—C21	1.459 (3)	C15—C16	1.398 (2)
N3—C10	1.289 (2)	C15—H15A	0.9300
N3—C8	1.389 (2)	C17—C18	1.506 (3)
C1—C6	1.391 (3)	C17—H17A	0.9700
C1—C2	1.398 (3)	C17—H17B	0.9700
C1—H1A	0.9300	C18—C19	1.518 (3)
C2—C3	1.390 (3)	C18—H18A	0.9700
C2—H2A	0.9300	C18—H18B	0.9700
C3—C4	1.392 (3)	C19—C20	1.512 (3)
C3—H3A	0.9300	C19—H19A	0.9700
C4—C5	1.388 (3)	C19—H19B	0.9700
C4—H4A	0.9300	C20—H20A	0.9700
C5—C6	1.392 (3)	C20—H20B	0.9700
C5—H5A	0.9300	C21—H21A	0.9600
C7—C8	1.374 (2)	C21—H21B	0.9600
C7—C22	1.478 (3)	C21—H21C	0.9600
C8—C9	1.454 (2)	C22—H22A	0.9600
C10—C11	1.463 (2)	C22—H22B	0.9600
C10—H10A	0.9300	C22—H22C	0.9600
			
C16—O2—C17	118.14 (15)	C14—C13—H13A	120.4
C9—N1—N2	109.46 (14)	C15—C14—C13	121.23 (17)
C9—N1—C6	124.67 (15)	C15—C14—H14A	119.4
N2—N1—C6	118.95 (15)	C13—C14—H14A	119.4
C7—N2—N1	107.41 (14)	C14—C15—C16	119.45 (17)
C7—N2—C21	121.40 (17)	C14—C15—H15A	120.3
N1—N2—C21	115.78 (16)	C16—C15—H15A	120.3
C10—N3—C8	118.86 (15)	O2—C16—C15	123.87 (16)
C6—C1—C2	118.8 (2)	O2—C16—C11	115.96 (15)
C6—C1—H1A	120.6	C15—C16—C11	120.17 (17)
C2—C1—H1A	120.6	O2—C17—C18	108.56 (15)
C3—C2—C1	120.3 (2)	O2—C17—H17A	110.0
C3—C2—H2A	119.9	C18—C17—H17A	110.0
C1—C2—H2A	119.9	O2—C17—H17B	110.0
C2—C3—C4	120.08 (19)	C18—C17—H17B	110.0
C2—C3—H3A	120.0	H17A—C17—H17B	108.4
C4—C3—H3A	120.0	C17—C18—C19	113.44 (16)
C5—C4—C3	120.3 (2)	C17—C18—H18A	108.9
C5—C4—H4A	119.9	C19—C18—H18A	108.9
C3—C4—H4A	119.9	C17—C18—H18B	108.9
C4—C5—C6	119.19 (18)	C19—C18—H18B	108.9
C4—C5—H5A	120.4	H18A—C18—H18B	107.7
C6—C5—H5A	120.4	C20—C19—C18	113.41 (18)
C1—C6—C5	121.36 (18)	C20—C19—H19A	108.9
C1—C6—N1	119.94 (18)	C18—C19—H19A	108.9
C5—C6—N1	118.69 (17)	C20—C19—H19B	108.9
N2—C7—C8	109.87 (15)	C18—C19—H19B	108.9
N2—C7—C22	121.27 (16)	H19A—C19—H19B	107.7
C8—C7—C22	128.82 (16)	C19—C20—I1	111.76 (15)
C7—C8—N3	122.69 (16)	C19—C20—H20A	109.3
C7—C8—C9	107.86 (15)	I1—C20—H20A	109.3
N3—C8—C9	129.42 (15)	C19—C20—H20B	109.3
O1—C9—N1	123.96 (16)	I1—C20—H20B	109.3
O1—C9—C8	131.28 (16)	H20A—C20—H20B	107.9
N1—C9—C8	104.73 (14)	N2—C21—H21A	109.5
N3—C10—C11	120.79 (16)	N2—C21—H21B	109.5
N3—C10—H10A	119.6	H21A—C21—H21B	109.5
C11—C10—H10A	119.6	N2—C21—H21C	109.5
C12—C11—C16	118.64 (16)	H21A—C21—H21C	109.5
C12—C11—C10	121.71 (16)	H21B—C21—H21C	109.5
C16—C11—C10	119.52 (16)	C7—C22—H22A	109.5
C13—C12—C11	121.30 (17)	C7—C22—H22B	109.5
C13—C12—H12A	119.3	H22A—C22—H22B	109.5
C11—C12—H12A	119.3	C7—C22—H22C	109.5
C12—C13—C14	119.12 (18)	H22A—C22—H22C	109.5
C12—C13—H13A	120.4	H22B—C22—H22C	109.5
			
C9—N1—N2—C7	−8.6 (2)	C6—N1—C9—O1	−21.4 (3)
C6—N1—N2—C7	−160.89 (17)	N2—N1—C9—C8	6.5 (2)
C9—N1—N2—C21	−147.91 (19)	C6—N1—C9—C8	156.82 (17)
C6—N1—N2—C21	59.8 (2)	C7—C8—C9—O1	175.93 (19)
C6—C1—C2—C3	−0.1 (4)	N3—C8—C9—O1	−6.2 (3)
C1—C2—C3—C4	0.0 (4)	C7—C8—C9—N1	−2.11 (19)
C2—C3—C4—C5	0.8 (3)	N3—C8—C9—N1	175.74 (17)
C3—C4—C5—C6	−1.4 (3)	C8—N3—C10—C11	−173.62 (16)
C2—C1—C6—C5	−0.5 (3)	N3—C10—C11—C12	7.8 (3)
C2—C1—C6—N1	−179.6 (2)	N3—C10—C11—C16	−176.25 (17)
C4—C5—C6—C1	1.3 (3)	C16—C11—C12—C13	−1.8 (3)
C4—C5—C6—N1	−179.60 (17)	C10—C11—C12—C13	174.17 (18)
C9—N1—C6—C1	−116.1 (2)	C11—C12—C13—C14	−0.9 (3)
N2—N1—C6—C1	31.7 (3)	C12—C13—C14—C15	2.6 (3)
C9—N1—C6—C5	64.8 (3)	C13—C14—C15—C16	−1.6 (3)
N2—N1—C6—C5	−147.45 (18)	C17—O2—C16—C15	1.2 (3)
N1—N2—C7—C8	7.2 (2)	C17—O2—C16—C11	−179.42 (17)
C21—N2—C7—C8	143.72 (19)	C14—C15—C16—O2	178.16 (18)
N1—N2—C7—C22	−170.71 (17)	C14—C15—C16—C11	−1.2 (3)
C21—N2—C7—C22	−34.2 (3)	C12—C11—C16—O2	−176.58 (16)
N2—C7—C8—N3	178.79 (16)	C10—C11—C16—O2	7.4 (2)
C22—C7—C8—N3	−3.5 (3)	C12—C11—C16—C15	2.8 (3)
N2—C7—C8—C9	−3.2 (2)	C10—C11—C16—C15	−173.23 (17)
C22—C7—C8—C9	174.55 (19)	C16—O2—C17—C18	−177.32 (16)
C10—N3—C8—C7	178.99 (17)	O2—C17—C18—C19	−55.7 (2)
C10—N3—C8—C9	1.4 (3)	C17—C18—C19—C20	−175.30 (18)
N2—N1—C9—O1	−171.71 (17)	C18—C19—C20—I1	67.8 (2)

### Hydrogen-bond geometry (Å, °)

**Table:**

*D*—H···*A*	*D*—H	H···*A*	*D*···*A*	*D*—H···*A*
C10—H10A···O1	0.93	2.30	2.995 (2)	132
C17—H17B···O1^i^	0.97	2.42	3.193 (2)	137

Symmetry codes: (i) *x*, −*y*+1/2, *z*−1/2.
